# The Use of Energy-Based Devices for the Treatment of Stress Urinary Incontinence: A Systematic Review and Meta Analysis of the Randomised Sham-Controlled Trials

**DOI:** 10.1007/s00192-026-06604-9

**Published:** 2026-03-19

**Authors:** David Lukanović, Geetika Shah, Miha Matjašič, Christian Phillips

**Affiliations:** 1 Department of Gynecology, Division of Gynecology and Obstetrics, Ljubljana University Medical Center, 1000 Ljubljana, Slovenia; 2 Department of Gynecology and Obstetrics, Faculty of Medicine, University of Ljubljana, 1000 Ljubljana, Slovenia; 3 Hampshire Hospitals, Urogynaecology, Basingstoke, UK; 4 Department of Education Studies, Faculty of Education, University of Ljubljana, 1000 Ljubljana, Slovenia

**Keywords:** Stress urinary incontinence, Energy-based device, Vaginal laser, Er:YAG laser, CO_2_ laser, Radiofrequency

## Abstract

**Introduction and Hypothesis:**

Energy-based devices (EBDs), including vaginal laser and radiofrequency therapies, have been proposed as minimally invasive treatments for stress urinary incontinence (SUI), but evidence remains limited and inconsistent. We hypothesised that EBDs would provide greater symptom improvement than sham, particularly in women with mild to moderate SUI.

**Methods:**

Following PRISMA guidelines, we searched Medline, Embase, the Cochrane Library, and One Search for randomised controlled trials (RCTs) comparing EBDs with sham in women with SUI, with at least 1 month of follow-up. The primary outcome was change in International Consultation on Incontinence Questionnaire–Urinary Incontinence Short Form (ICIQ-UI SF) scores. Two reviewers independently performed data extraction and RoB-2 assessment. Random-effects meta-analyses using restricted maximum likelihood estimation were conducted.

**Results:**

Ten RCTs (11 datasets; ~ 850 women) were included. Using a random-effects model with Knapp–Hartung adjustment, the pooled EBD analysis showed a mean difference of –1.08 points (95% CI –2.08 to –0.08), indicating a statistically significant improvement with EBD. The prediction interval (–3.63 to 1.48) suggests a wide range of possible effects, including no benefit. Heterogeneity was moderate (I^2^ = 53%). Subgroup analysis of non-ablative Er:YAG laser showed the strongest and most homogeneous effect (MD –1.42; 95% CI –2.55 to –0.28; I^2^ = 29%). CO_2_ laser findings were inconsistent, and evidence for radiofrequency was insufficient. Adverse events were mild and transient.

**Conclusions:**

EBDs may improve SUI symptoms compared with sham, with the most consistent benefit observed for non-ablative Er:YAG laser. However, effects are modest and short-term. High-quality RCTs with standardised protocols and long-term follow-up are needed.

**Supplementary Information:**

The online version contains supplementary material available at https://doi.org/10.1007/s00192-026-06604-9.

## Introduction

Urinary incontinence (UI) affects approximately 44–57% of middle-aged and postmenopausal women and can have significant physical, psychological, and social consequences [1]. Stress urinary incontinence (SUI) is defined as “the complaint of involuntary leakage on effort or exertion, or on sneezing or coughing” [2]. In women, the primary contributing factors include age-related weakening of the pelvic floor and childbirth, which can result in damage to the connective tissues supporting the bladder and urethra. Collagen, comprising up to 80% of the protein content in the endopelvic fascia, plays a key structural role in these tissues [3]. Reduced collagen levels have been observed in pelvic tissues of women with SUI [4]. Laser treatments aim to strengthen the suburethral connective tissue (“hammock”) to improve continence.

First-line management of SUI typically includes conservative approaches such as lifestyle changes, pelvic floor muscle training, and pessary use [5]. When these are insufficient, surgical interventions such as tension-free vaginal tape (TVT), bulking agents, colposuspension, or autologous fascial slings are considered. Although effective, surgery carries risks of adverse events, some of which may be serious and life-altering. Increasingly, patients are seeking and preferring less invasive options that offer quicker recovery and reduced morbidity, despite a potential lower efficacy compared to more major surgical options [6].

The first laser (ruby laser) was developed in the 1960 s and widely adopted in medicine and gynaecology by the 1980 s [7] for ablation and excision of tissue. However, the technology has evolved and evidence now demonstrates the photothermal effects of laser can promote tissue remodelling. These photothermal effects stimulate neocollagenesis, collagen contraction, and tissue tightening with minimal adverse effects [3, 8]. The CO_2_ and Er:YAG lasers are the two main types used in gynaecology, utilising either ablative or non-ablative modes of action to induce collagen reorganisation via different mechanisms [3]. Similarly, radiofrequency (RF) devices, which emit monopolar or bipolar electromagnetic waves, produce deep tissue heating and can be delivered via vaginal or transurethral routes.

Although previous reviews have examined EBDs in SUI, they often suffer from heterogeneity in study design, follow-up, outcome measures and limited numbers of randomised controlled trials (RCTs). To support safe and evidence-based clinical implementation of the technology into routine practice there is a need for an updated and focused analysis [3, 9–11].

The aim of this study is to conduct a systematic review and meta-analysis evaluating the efficacy and safety of energy-based devices, including laser and radiofrequency technologies, in the treatment of SUI in women. Specifically, we aim to assess outcomes soley from RCTs comparing EBDs to sham treatments, and provide an evidence base to guide clinical use and future research in this emerging field.

## Materials and Methods

This systematic review and meta-analysis were conducted following the Cochrane Handbook for Systematic Reviews of Interventions [12] and adhered to the Preferred Reporting Items for Systematic Reviews and Meta-Analyses (PRISMA) guidelines [13]. The study protocol was registered in the International Prospective Register of Systematic Reviews (PROSPERO) database. Ethical approval was not required due to the nature of the study design.

### Eligibility Criteria, Information Sources, and Search Strategy

A comprehensive literature search was performed across multiple electronic databases, including PubMed, Embase, Cochrane Central Register of Controlled Trials (CENTRAL), Web of Science, Scopus, and the University of Winchester's One Search engine, which aggregates content from sources such as EBSCOhost, Ingenta Connect, Gale Academic OneFile, and others. The search spanned publications up to 1 December 2025.

The search strategy employed both keywords and Medical Subject Headings (MeSH) terms related to stress urinary incontinence (SUI) and energy-based devices (EBDs), including: “urinary incontinence,” “stress urinary incontinence,” “energy-based devices,” “laser therapy,” “radiofrequency,” “CO_2_ laser,” and “Er:YAG laser.” Boolean operators were applied to refine the search. The specific search strategies for each database are detailed in Supplementary Material 1.

### Study Selection

Studies were considered eligible for inclusion if they investigated adult women aged 18 years or older with a diagnosis of stress urinary incontinence (SUI). The intervention of interest was treatment with energy-based devices, including CO_2_ lasers, Er:YAG lasers, or radiofrequency devices. Only randomised controlled trials (RCTs) published in English were included, and all studies were required to have a minimum follow-up period of 1 month post-treatment. Importantly, only studies that used a sham control group were included.

Studies were excluded if they were not an RCT, included participants under the age of 18, did not specifically focus on SUI, or failed to report relevant outcome data. Trials that compared energy-based devices with physiotherapy/pelvic floor muscle training, or other vaginal therapies were also excluded, as were publications not available in English, letters, and non–peer-reviewed sources.

Two independent reviewers screened the titles and abstracts of identified studies. Full texts of potentially eligible articles were retrieved and assessed for inclusion. Discrepancies were resolved through discussion with a third reviewer.

## Outcomes

The primary outcome was improvement in SUI symptoms, measured with validated instruments, primarily the International Consultation on Incontinence Questionnaire–Urinary Incontinence Short Form (ICIQ-UI SF). As the ICIQ-UI SF is the most widely validated questionnaire, our analysis focused exclusively on this tool.

### Data Extraction

Data extraction was conducted using standardised forms. Extracted information included study characteristics (e.g. author, year, country), participant demographics, intervention details (e.g. type of EBD, treatment protocol), outcome measures, results, and adverse events. One reviewer performed the extraction, and a second reviewer verified the accuracy. Any disagreements were resolved through consensus with a third reviewer.

### Assessment of Risk of Bias

The risk of bias in included studies was evaluated using the Cochrane Risk of Bias 2 (RoB 2) tool for randomised trials [14]. This tool assesses bias across five domains: randomisation process, deviations from intended interventions, missing outcome data, measurement of the outcome, and selection of the reported result. Each domain was rated as having a low, some concerns and high risk, or without any information/unclear risk of bias. Assessments were conducted independently by two reviewers, with disagreements resolved through discussion with a third reviewer.

### Data Synthesis

All statistical analyses were performed using R (version 4.2.3, R Foundation for Statistical Computing, Vienna, Austria). Meta-analyses were implemented with metaphor package, with dplyr, tidyr, tibble, and readr packages for data management and ggplot2 package for figures. Moreover the effect measure was the mean difference (MD) on the ICIQ-SF scale, oriented as post—baseline, such that negative values favour the active intervention.

When studies reported change scores with their variability, those values were used directly. When only pre- and post-treatment means and SDs were available, the SD of change was derived as *SD*_Δ_ = $\sqrt{{SD}_{pre}^{2}+ {SD}_{post}^{2}-2\mathrm{r}\times{SD}_{pre}\times{SD}_{post}}$ with a primary pre–post correlation *r* = 0.50 and sensitivity analyses at *r* = 0.30 and *r* = 0.70. For studies reporting medians and interquartile ranges (IQRs), means and SDs were approximated using standard normal-based conversions (mean = (*Q*1 + median + *Q*3)/3; SD = (*Q*3 − *Q*1)/1.35 * (Q3 − Q1)/1.35). Furthermore pooled effects were estimated with a random-effects model using restricted maximum likelihood (REML) for τ^2^. To improve small-sample inference, we applied the Knapp–Hartung (KH) adjustment for tests and 95% confidence intervals of the pooled MD. Studies were weighted by inverse variance under the random-effects model. We additionally report a 95% prediction interval. Publication bias was evaluated using funnel plots and Egger’s regression test.

## Results

### Study Selection

**Fig. 1:**
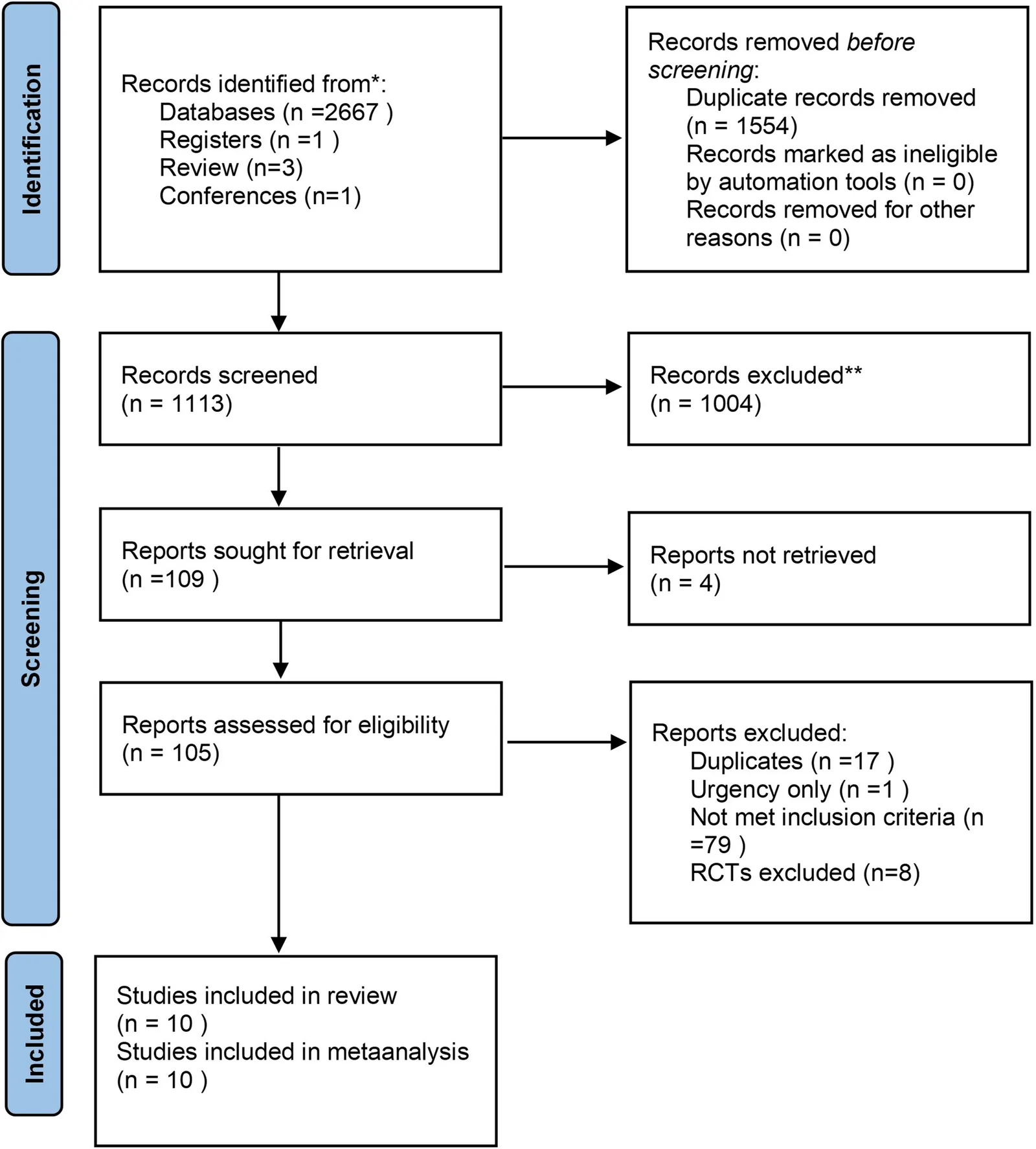
Flow diagram of evidence acquisition in the systematic review and metanalysis

We identified 2667 studies from databases (PubMed, Embase, and others), one record from trial registries, three from review articles, and one from conference abstracts (Fig. 1). After screening and eligibility assessment, 10 randomised controlled trials met the inclusion criteria and were included in the systematic review and meta-analysis. Importantly, eight RCTs were excluded because they lacked a sham control group, using physiotherapy or vaginal lubricants as comparators instead and one RCT was excluded as it did not have any follow-up after the treatment. Notably, one included study evaluated two different energy-based devices (CO_2_ laser and radiofrequency) versus sham, and was therefore analysed as two separate datasets.

### Risk of Bias in Studies and Levels of Evidence

**Fig. 2:**
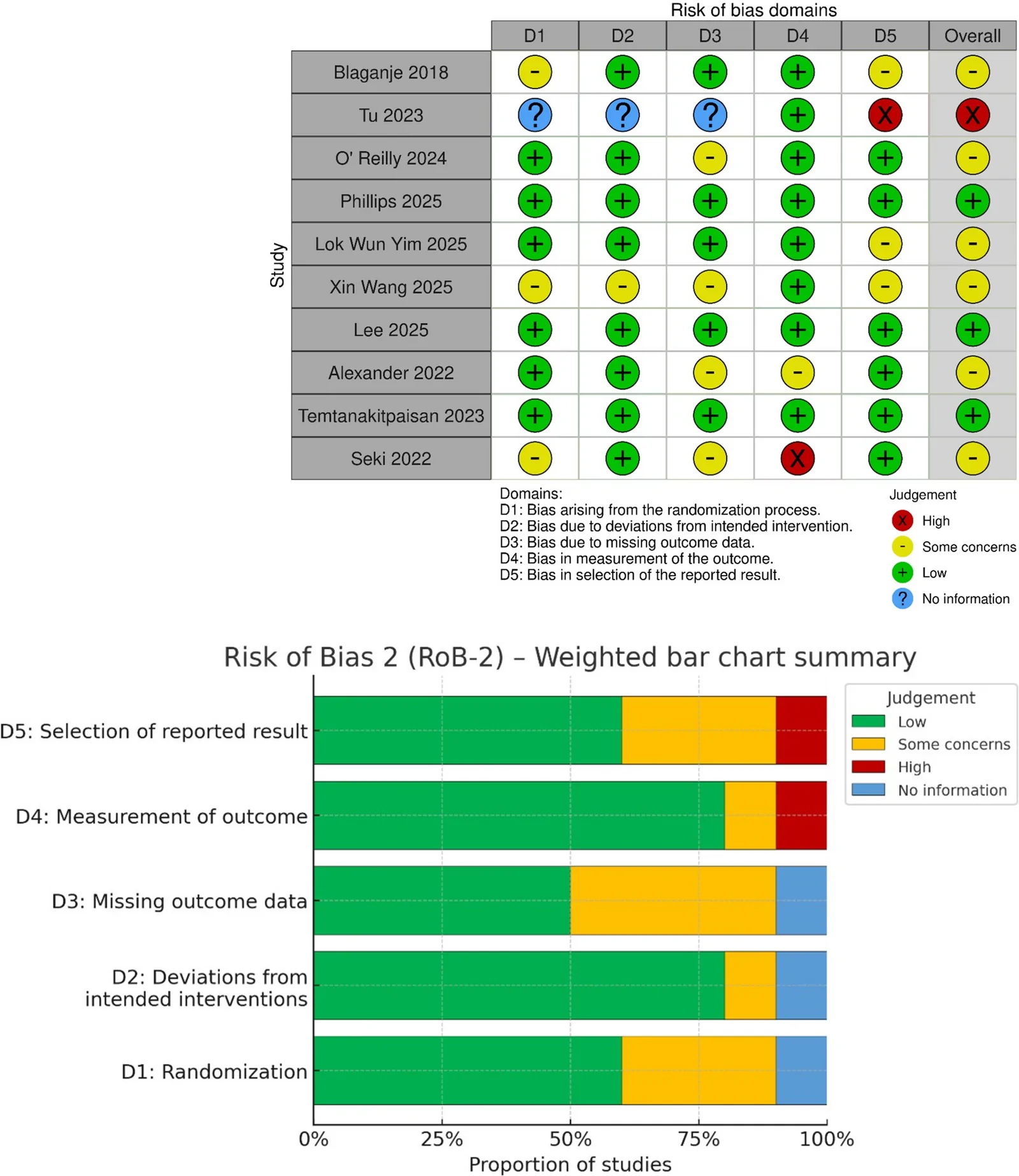
Risk of bias analysis of the 10 randomised sham-control trials [15]. **a:** Continued risk of bias analysis of the 10 randomised sham-control trials [15]

Risk of bias was assessed with the Cochrane RoB-2 tool. Most trials were at low risk for deviations from intended interventions, missing data, and outcome measurement, while some concerns were noted in randomisation and selective reporting. One study (Tu 2023) was judged high risk due to limited reporting, and another (Seki 2022) showed concerns about selective outcome reporting. The weighted bar chart (Fig. 2) confirms that over 70% of studies were low risk in most domains, with 30–40% showing some concerns in randomisation and reporting.

### Study Characteristics and Study Results

A total of 10 randomised controlled trials were included, with one study [25] contributing two treatment arms (CO_2_ laser and radiofrequency), resulting in 11 datasets overall (Table 1). Across trials, sample sizes ranged from 55 to 144 participants, with most including between 60 and 120 women.

**Table 1:** Main characteristics of the studies incorporated in the SR

Author and year	Type of EBD used	Number of treatments	Time between treatments	Follow-up post treatment	Sample size		Type of UI	Severity of SUI
Blaganje et al., 2018 [16]	Non-ablative ErYAG, Fotona doo, Slovenia	1	/	3 moths	Laser	56	SUI	Mild, moderate and severe
					Sham	56		
O' Reilly et al., 2024 [17]	Non-ablative ErYAG, Fotona doo, Slovenia	2	1 month	6, 12 months	Laser	71	SUI	Mild, moderate and severe
					Sham	37		
Phillips et al., 2025 [18]	Non-ablative ErYAG, Fotona doo, Slovenia	3	4–6 weeks	6 months	Laser	51	SUI	Mild to moderate
					Sham	23		
Lok Wun Yim et al., 2025 [19]	Ablative Er:YAG, Juliet, Jena, Germany	2	4 weeks	6, 12 months	Laser	52	SUI and MUI (predominat SUI)	Mild, moderate and severe
					Sham	23		
Xin Wang, et al., 2025 [20]	Non-ablative ErYAG, Fotona doo, Slovenia	3	1 moths	1, 3, 6 months	Laser	84	SUI	Mild to moderate
					Sham	42		
Lee et al., 2025 [21]	Non-ablative ErYAG, Fotona doo, Slovenia	2	6 weeks	6 weeks, 6 months	Laser	76	SUI	Mild, moderate, mostly severe and very severe
					Sham	68		
Tu et al., 2023 [22]	Non-ablative ErYAG, Fotona doo, Slovenia	2	4 weeks	6, 12 months	Laser	20	SUI and MUI (predominat SUI with ISD)	Mild to moderate SUI + patients with ISD (so moderate to severe spectrum represented
					Sham	15		
Alexander et al., 2022 [23]	MonaLisa Touch® fractional microablative CO_2_ laser system (DEKA, Florence, Italy)	1	/	3 months	Laser	47	SUI	No severity recorded
					Sham	42		
Temtanakitpaisan et al., 2023 [24]	MonaLisa Touch® fractional microablative CO_2_ laser system (DEKA, Florence, Italy)	3	4–6 weeks	3 months	Laser	30	SUI	Mild to moderate
					Sham	29		
Seki et al., 2022 [25]	CO_2_ laser (Alma-Femilift™)	3	4 weeks	1, 6, 12, 18, 24, 30, 36, 42, 48 months	Laser	38	SUI	Mild to moderate
	Radiofrequency (Alma-Vaginal RF device)	3	4 weeks	1, 6, 12, 18, 24, 30, 36, 42, 48 months	RF	38		
					Sham	38		

The majority of studies investigated Er:YAG laser therapy [16–22], while three trials evaluated fractional microablative CO_2_ lasers [23–25] and one evaluated radiofrequency therapy [25]. All studies used sham controls, and treatment protocols typically consisted of 2–3 sessions administered 4–6 weeks apart, except Blaganje et al. [16] and Alexander et al. [23], which used a single laser session.

Follow-up periods were most commonly 6 months, with some extending to 12 months [17, 22]. The International Consultation on Incontinence Questionnaire – Short Form (ICIQ-SF) was the most frequently used primary outcome measure, reported in all Er:YAG studies and several CO_2_ studies. Regarding participants, most trials included women with mild-to-moderate stress urinary incontinence (SUI), though Tu et al. [22] additionally enrolled women with stress-predominant mixed UI and intrinsic sphincter deficiency (ISD). Baseline severity was generally defined by ICIQ-SF scores, though one study [23] did not specify detailed severity categories. Table 1 demostrates the majorty of studies included patients predominantly with mild to moderate SUI but some had a significant proportion of women with severe SUI.

### Results of Synthesis

The meta-analysis included 10 randomised controlled trials (11 datasets) evaluating the effectiveness of energy-based devices (EBDs) for stress urinary incontinence (SUI), with the ICIQ-UI SF score as the primary outcome. Patient-reported outcomes were analysed as mean differences in score changes from baseline to follow-up, with negative values indicating improvement in the active treatment group. For studies that did not report variability for change scores, standard deviations were imputed assuming a pre–post correlation of 0.5. Sensitivity analyses using correlations of 0.3 and 0.7 yielded similar results, confirming robustness.

**Fig. 3:**
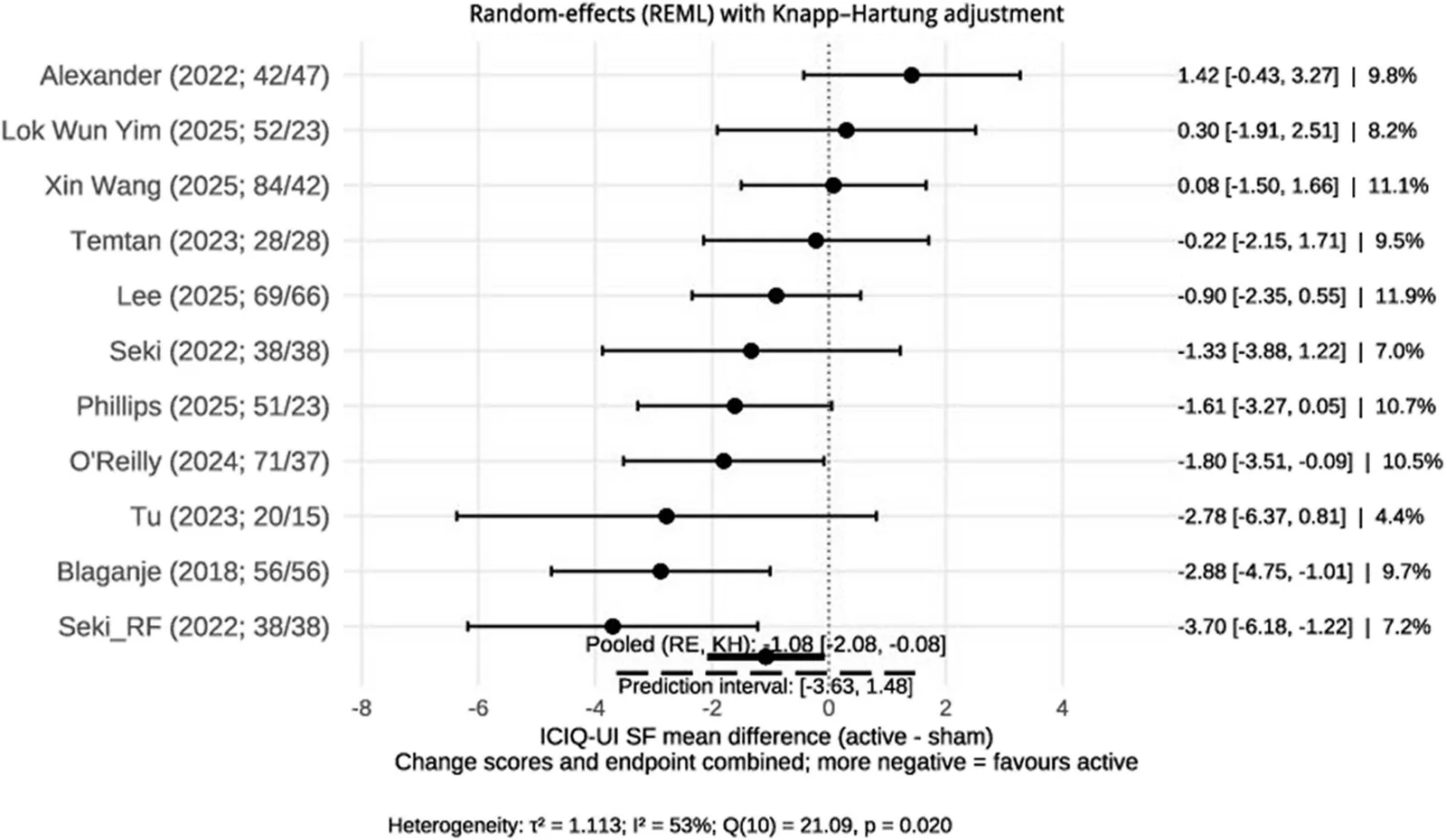
Forest plot of RCTs comparing EBDs versus sham for SUI, using ICIQ-UI SF mean differences between baseline and follow-up. Random-effects meta-analysis (REML) with Knapp–Hartung adjustment of the mean difference (MD) in change on the ICIQ-UI SF comparing EBD with sham (post–baseline, negative favours active treatment). Points and horizontal bars show study-level MDs with their 95% CIs; right-hand text reports each MD [95% CI] and the corresponding random-effects weight. The thick bar and dot indicate the pooled random-effects estimate with its 95% CI (KH), while the long dashed line represents the prediction interval. Heterogeneity statistics (τ^2^, I^2^, and Q-test) are reported below the plot. The Lok Wun Yim (2025) dataset required median/IQR to mean/SD conversion (asterisk), and the Seki_RF (2022) study represents a radiofrequency modality rather than laser

Using a random-effects model with Knapp–Hartung adjustment, the pooled mean difference was –1.08 (95% CI –2.08 to –0.08), indicating a statistically significant improvement with EBD compared with sham (Fig. 3). The prediction interval (–3.63 to 1.48) suggests a broad range of possible effects in future studies, including the possibility of no benefit. Moderate heterogeneity was observed (I^2^ = 53%, τ^2^ = 1.113; Q(10) = 21.09, *p* = 0.020), reflecting clinical and methodological variability across trials.

After exclusion of the radiofrequency (RF) trial, 10 datasets evaluating laser therapies (CO_2_ and Er:YAG) versus sham were included in a second random-effects meta-analysis. Several studies—including O’Reilly (2024), Blaganje (2018), Phillips (2025), and Tu (2023)— demonstrated the largest reductions in ICIQ-UI SF scores favouring active treatment, whereas others reported minimal or no difference. The pooled mean difference was –0.87 (95% CI –1.81 to 0.07). Although the point estimate continued to favour laser therapy, the confidence interval crossed zero, indicating loss of statistical significance once the RF dataset was removed. The prediction interval (–3.04 to 1.30) indicates that future studies may show effects ranging from clinically important benefit to no difference. Heterogeneity was moderate (I^2^ = 45%, τ^2^ = 0.747; Q(9) = 16.34, *p* = 0.060) and lower than in the full model, suggesting that RF contributed to between-study variability.

**Fig. 4:**
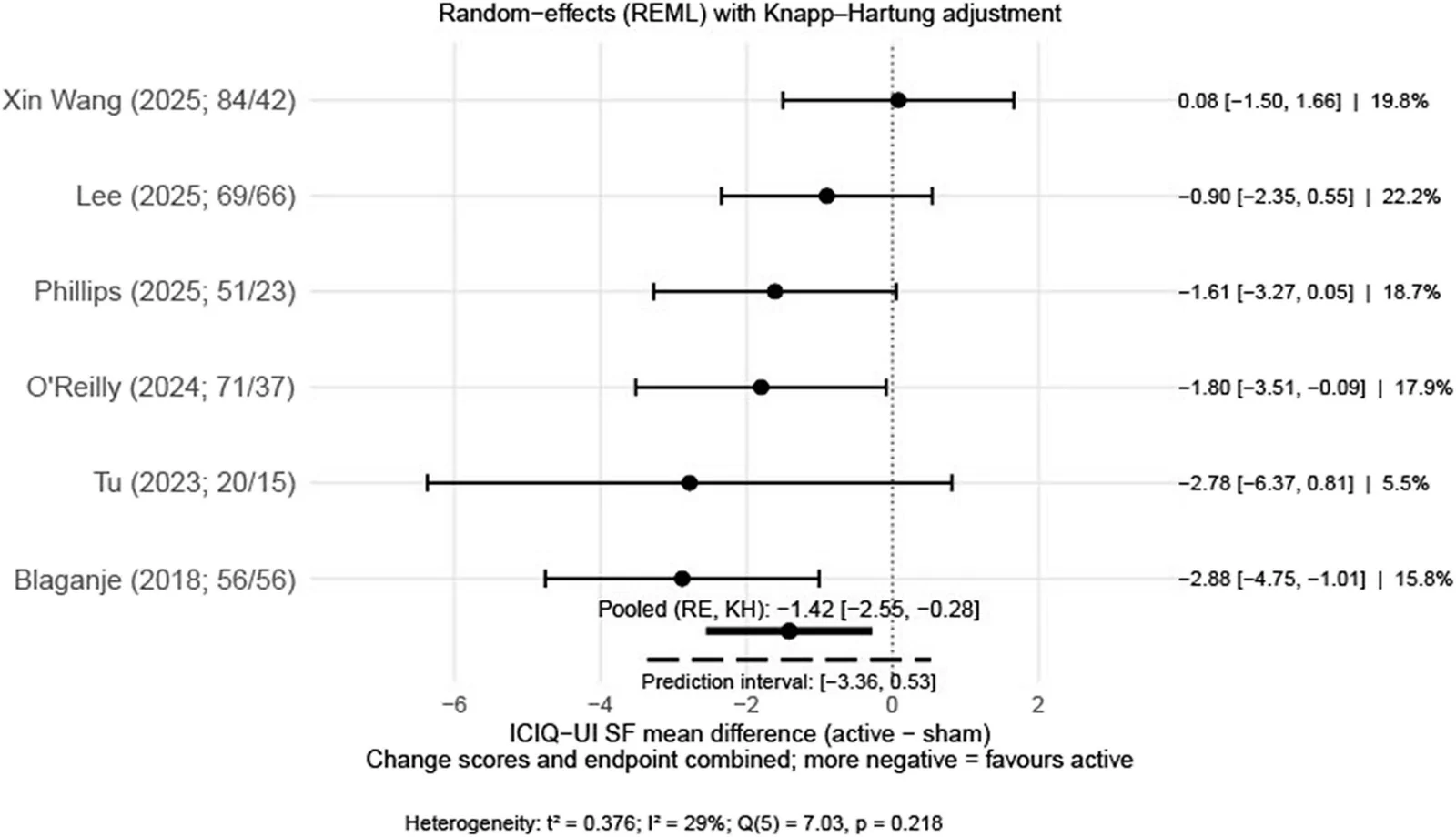
Forest plot of RCTs comparing non-ablative Er:YAG laser versus sham for SUI, using ICIQ-UI SF mean differences between baseline and follow-up. Random-effects meta-analysis (REML) with Knapp–Hartung adjustment of the mean difference (MD) in ICIQ-UI SF change (negative values favour active treatment). Points and horizontal bars represent study-level MDs with 95% CIs; the right-hand text reports each MD [95% CI] and its random-effects weight. The thick bar and dot indicate the pooled random-effects estimate with its 95% CI (KH), and the long dashed line represents the prediction interval. Heterogeneity statistics (τ^2^, I^2^, Q-test) are shown below. The analysis includes only studies using a non-ablative Er:YAG mechanism of action; the ablative-mode Er:YAG study (Lok Wun Yim, 2025) was excluded due to differing MOA

A further subgroup analysis focused on six studies that all used the same non-ablative Er:YAG laser, enabling assessment under a uniform mechanism of action (Fig. 4). The study by Lok Wun Yim was excluded because it used an ablative (“cold ablation”) mode, which does not share the same photothermal non-ablative mechanism. Individual study effects again varied, with Blaganje (2018), O’Reilly (2024), and Phillips (2025) showing the most consistent and meaningful improvements. Using a random-effects model with Knapp–Hartung adjustment, the pooled mean difference was –1.42 (95% CI –2.55 to –0.28), demonstrating a statistically significant benefit of non-ablative Er:YAG compared with sham. The prediction interval (–3.36 to 0.53) suggests that while most future studies using this modality are expected to show improvement, some may observe little or no effect. Heterogeneity was low to moderate (I^2^ = 29%, τ^2^ = 0.376; Q(5) = 7.03, *p* = 0.218), indicating greater consistency when analyses were restricted to a homogeneous technology and mechanism of action.

Across individual trials, Blaganje et al. [16] and O’Reilly et al. [17] demonstrated the largest and most precise improvements with non-ablative Er:YAG. Phillips et al. [18] also favoured active treatment, though with smaller effect sizes and confidence intervals that included the null. The small study by Tu et al. [22] showed a large but imprecise effect, reflecting limited sample size. Results from Lok Wun Yim et al. [19] should be interpreted with caution, as means and standard deviations were derived from medians and interquartile ranges and because the ablation-based mode of action differs from the non-ablative mechanism used in the six Er:YAG studies included in the subgroup analysis.

## Discussion

This systematic review and meta-analysis, which included 11 datasets from 10 randomised controlled trials of EBDs for SUI, showed a statistically significant reduction in ICIQ-UI SF scores in the active group compared with sham (mean difference –1.08 points; 95% CI –2.08 to –0.08). Although the effect size was modest, the result was statistically significant, and moderate heterogeneity was present (I^2^ = 54%). The wide prediction interval (–3.63 to 1.48) indicates that while many women may experience clinically meaningful improvement, some may derive little or no benefit. Current evidence has led many professional societies to recommend that vaginal lasers be used only within research settings rather than in routine clinical practice, given the lack of large, well-designed randomised controlled trials [26–29]. This highlights the importance of systematic reviews and meta-analyses in guiding cautious, stepwise implementation of EBD therapy for SUI.

The recent Cochrane Review by Ippolito et al. [9] provides crucial context. Analysing data from nine trials (689 women), it similarly reported a small advantage of vaginal laser therapy over sham or control in the short term (mean difference –1.42; 95% CI –2.41 to –0.43; I^2^ = 54%), but with very low-certainty evidence. Notably, for CO_2_ lasers, the pooled effect (–1.25; 95% CI –3.57 to 1.07) was inconclusive, with high heterogeneity (I^2^ = 68%). In contrast, Er:YAG lasers yielded a statistically significant benefit (–1.60; 95% CI –2.63 to –0.57) with moderate heterogeneity (I^2^ = 36%). Despite this, Ippolito et al. [9] emphasised that the observed effects did not meet the minimally important clinical difference, and the overall certainty of evidence was very low.

Our findings align closely with those of Ippolito et al. [9]. The findings of both reviews (Ippolito and ours) suggest that non-ablative Er:YAG laser has the most consistent results demonstrating benefit, while CO_2_ laser data remain inconsistent. Several trials [16–18] demonstrated improvements, whereas others [20, 23] did not. This variability likely reflects differences in laser protocols, patient demographics (age, parity, menopausal status, BMI), and definitions of SUI severity—most of which were not standardised across studies. The severity SUI appears to play an important role in the effectiveness of EBDs. The RCTs to date that have specifically enrolled women with mild to moderate SUI were far more likely to demonstrate an improvement in symptoms compared with studies that had a higher proportion of women with severe SUI. In these populations, Er:YAG and CO_2_ lasers have demonstrated small but measurable reductions in ICIQ-UI SF scores compared with sham controls. Importantly, our extraction sub-analysis of non-ablative Er:YAG revealed the strongest and most homogeneous treatment signal, indicating a statistically significant and more consistent benefit. This sub-analysis indicates that non-ablative Er:YAG shows the most consistent and homogeneous treatment effect among the evaluated EBD modalities effect than any other subgroup examined.

In our review, we excluded RCTs, which did not provide any post-treatment follow-up data, such as the study by Hrefna B. Sigurðardóttir et al. [30], which reported only immediate outcomes. Similarly, trials that compared vaginal laser therapy to PFMT rather than to sham were excluded from the quantitative synthesis to reduce further heterogeneity. Notably, three recent RCTs have directly compared laser with PFMT: da Fonseca et al. [31], Rocha-Rangel et al. [32], and Page et al. [33]. Collectively, these studies found that PFMT achieved outcomes comparable to, or in some cases superior to, vaginal Er:YAG laser in women with SUI. Da Fonseca et al. [31] reported that PFMT resulted in greater improvement in pad test results, while Rocha-Rangel et al. [32] and Page et al. [33] observed no significant differences between groups, though both interventions led to symptom improvement. Overall, these findings reinforce that PFMT remains a highly effective, guideline-recommended first-line therapy, whereas the role of laser therapy should be considered adjunctive or experimental, pending further high-quality comparative data, although current evidence demonstrates benefits similar to PFMT. Given that PFMT targets the pelvic floor musculature, while laser therapy acts primarily on the endopelvic fascia and connective tissue, a combined approach may offer a promising non-surgical outpatient treatment. A trial assessing the combined use of non-ablative Er:YAG laser and magnetic stimulation, the latter aimed at inducing pelvic floor muscle contractions, is currently registered; results have not yet been reported [34]. Moreover, a recent RCT from Lunardi et al. [35] concluded that the combination of techniques (RF + PFMT) showed superior results for treating UI in climacteric women over 6 months, promoting improvement in urinary symptoms and pelvic floor function compared to isolated treatments [35]. The RCT by Lu et al. [36] further demonstrated that combined platelet-rich plasma (PRP) and fractional CO_2_ laser therapy significantly improved UI symptoms and quality of life compared with either treatment alone, suggesting that multimodal approaches may enhance outcomes in women with mild to moderate SUI.

Safety profiles across included trials were generally favourable. Most studies reported only minor, transient adverse events such as vaginal discharge, oedema, spotting, or discomfort. One notable side effect was abnormal uterine bleeding, which may be explained by contact bleeding from the vaginal probe in participants with atrophic vaginal epithelium. In some cases, side effects were reported only after the initial treatment, possibly reflecting improvements in vulvovaginal atrophy with repeated sessions. As Er:YAG laser is most frequently used, analysis of the MAUDE database has shown serious events are most often associated with ablative lasers, especially when treatments are performed injudiciously and outside treatment protocols/parameters [37]. A retrospective review of 113,000 patients treated with non-ablative Er:YAG showed adverse events were infrequent and no severe adverse events [18, 38].

Evidence on the long-term efficacy of laser therapy for SUI remains limited. One study reported that ICIQ-UI SF scores returned to baseline within 12–18 months [39]. Some data suggest that periodic “top-up” sessions every 6 months may help maintain symptom improvement [40, 41]. In addition, one RCT found that a single maintenance treatment provided continued benefit in women who had previously responded to laser therapy, indicating that maintenance sessions may be both necessary and effective [42]. The longest long-term follow-up to date by Seki et al. [43] reported that although continence rates declined over time, more than 50% of perimenopausal women with mild to moderate, pure or predominant SUI remained free of symptoms for at least 24 months after fractional CO_2_ laser or microablative RF treatment, with no major complications observed. Nevertheless, long-term efficacy remains uncertain and requires further study [43].

This review has several limitations. First, the number of RCTs remains small, restricting statistical power and limiting subgroup analysis by device type, SUI severity, or patient characteristics. Second, heterogeneity in EBD mode of action, study protocols, populations, and outcomes reduces generalisability. Third, most studies relied on subjective measures (ICIQ-UI SF), with fewer including objective outcomes such as pad tests or urodynamic studies. Fourth, follow-up was generally short-term (usually 6 months), with only a few trials reporting outcomes beyond one year. Fifth, evidence for RF remains insufficient. Only one sham-controlled RCT [25] directly compared RF to sham, reporting short-term improvement in ICIQ-UI SF scores. However, given the single-trial evidence base, small sample size, and methodological limitations, no firm conclusions can be drawn regarding the clinical effectiveness or durability of RF. At present, the data do not allow for meaningful synthesis or subgroup analysis, and further high-quality RCTs are required before RF can be considered a reliable therapeutic option for women with SUI. Sixth, this review included only studies published in the English language, which introduces a potential risk of language bias. Although English-language publications dominate the field and are typically published in internationally indexed, peer-reviewed journals with standardised reporting suitable for meta-analysis, relevant evidence published in other languages may have been missed. Finally, publication bias cannot be excluded, given the predominance of small, single-centre studies.

## Conclusion

This systematic review and meta-analysis indicates that EBDs may provide a modest short-term improvement in SUI compared with sham treatment. The overall effect of EBDs is small, inconsistent across devices/modalities and supported by trials with methodological limitations and short follow-up. Importantly, the subgroup analysis of non-ablative Er:YAG laser demonstrated statistically significant reduction in ICIQ-SF and the most consistent and homogeneous treatment effect. EBDs appear safe with adverse events mostly mild and transient. Future large, well-designed RCTs with standardised protocols, objective outcomes, and long-term follow-up are essential to define EBD’s role in the management of SUI.

## Supplementary Information

Below is the link to the electronic supplementary material.

Supplementary file1 (DOCX 15.0 KB)

## Data Availability

All data analyzed during this study are included in the published articles cited in this review.
