# Supplementary material for: The Use of Energy-Based Devices for the Treatment of Stress Urinary Incontinence: A Systematic Review and Meta Analysis of the Randomised Sham-Controlled Trials
Source: Int Urogynecol J. 2026 Mar 19;37(9):2779–90. doi: 10.1007/s00192-026-06604-9 (PMC13649917; doi:10.1007/s00192-026-06604-9)
Supplement: Supplementary file 1 [file 192_2026_6604_MOESM1_ESM.docx]

Search strategy

| Search engine/ Data base | Search strategy |
| --- | --- |
| PubMed,  Cochrane Central Register of Controlled Trials (CENTRAL),  Web of Science,  Scopus,  Embase,  One search^*^ | Concept 1:  Laser* OR “laser therapy” OR “laser therapies” OR “pulsed-laser” OR “Lasers, Solid-State” OR “Solid-State-Laser” OR “Yttrium Aluminium Garnet” OR “Erbium-YAG” OR YAG OR “CO2-laser” OR “carbon dioxide laser” OR “energy devices” OR “radiofrequency”.  AND  Concept 2:  “Urinary Incontinence” OR “urinary stress incontinence” OR “stress incontinence” OR “urinary urge incontinence”  NOT    Concept 3:  Prostate |

*EBSCOhost electronic journals service, EBSCOhost e-journals, Scopus, Ingenta connect, Ingenta connect journals, Gale Academic OneFile, Elsevier Sciencedirect freedom collection journals, EBSCOhost Medline complete, KB+ Jisc collections Wiley online library, Gale General OneFile, Springerlink journals- auto holdings, MCLS Springerlink Journals, Springer online journal DRAA, Westlaw international journals.
